# High prevalence of advanced colorectal neoplasia in the Thai population: a prospective screening colonoscopy of 1,404 cases

**DOI:** 10.1186/s12876-016-0526-0

**Published:** 2016-08-23

**Authors:** Bunchorn Siripongpreeda, Chulabhorn Mahidol, Navara Dusitanond, Tassanee Sriprayoon, Bunlung Muyphuag, Thaniya Sricharunrat, Narongchai Teerayatanakul, Watanya Chaiwong, Wipra Worasawate, Prassanee Sattayarungsee, Juthamas Sangthongdee, Jirapa Prarom, Gaidganok Sornsamdang, Kamonwan Soonklang, Kasiruck Wittayasak, Chirayu U. Auewarakul

**Affiliations:** 1 Chulabhorn Hospital, Bangkok, Thailand; 2 Chulabhorn Research Institute, Bangkok, Thailand; 3 Faculty of Medicine Siriraj Hospital, Mahidol University, Bangkok, Thailand

**Keywords:** Colorectal cancer, Adenoma, Advanced colorectal neoplasia, Screening colonoscopy, Fecal immunochemical test, National policy

## Abstract

**Background:**

Increasing morbidity and mortality from colorectal cancer is evident in recent years in the developing Asian nations. Particularly in Thailand and most neighbouring low-income countries, screening colonoscopy is not yet recommended nor implemented at the national policy level.

**Methods:**

Screening colonoscopy was offered to 1,500 healthy volunteers aged 50–65 years old who were registered into the program between July 2009 and June 2010. Biopsy and surgery was performed depending on the identified lesions. Fecal immunochemical tests (FIT) were additionally performed for comparison with colonoscopy.

**Results:**

There were 1,404 participants who underwent colonoscopy. The mean age of the cohort was 56.9 ± 4.2 years and 69.4 % were females. About 30 % (411 cases) of all colonoscopies had abnormal colonoscopic findings, and of these, 256 cases had adenomatous polyps. High risk adenomas (villous or tubulovillous or high grade dysplasia or size > 1 cm or > 3 adenomatous polyps) were found in 98 cases (7 %), low risk adenoma in 158 cases (11.3 %), and hyperplastic polyps in 119 cases (8.5 %). Eighteen cases (1.3 %) had colorectal cancer and 90 % of them (16 cases) were non-metastatic including five stage 0 cases, seven stage I cases, and four stage IIA cases. Only two cases had metastasis: one to regional lymph nodes (stage IIIB) and another to other organs (stage IVA). The most common cancer site was the distal intestine including rectum (7 cases, 38.9 %) and sigmoid colon (7 cases, 38.9 %). Ten colorectal cancer cases had positive FIT whereas 8 colorectal cancer cases were FIT-negative. The sensitivity and specificity of FIT was 55.6 % and 96.2 %, respectively, while the positive predictive value was 16.4 % and negative predictive value was 99.4 %. The overall survival of colorectal cancer cases at 5-year was 83.3 %.

**Conclusion:**

High prevalence of colorectal cancer and high-risk adenoma was found in the Thai population aged 50–65 years old by screening colonoscopy. FIT was not sensitive enough to detect colorectal cancer in this asymptomatic cohort. Integration of screening colonoscopy into the national cancer screening program should be implemented to detect early cases of advanced colorectal neoplasia and improve survival of colorectal cancer patients in Thailand.

## Background

Colorectal cancer is one of the most common causes of cancer death globally each year, along with lung, liver, prostate, and breast cancer [1]. Disease incidence of economically developed countries in Asia such as Japan, Korea and Singapore abruptly approached the level of the Western countries. Data from the National Cancer Institute of Thailand Cancer Registry also showed a gradual increase in the number of new colorectal cancer patients in recent years with increased advance stage cases identified [2]. Colorectal cancer screening can diagnose cases in early stages, decrease cancer mortality and potentially prevent this disease [3, 4]. Detection and proper management of advanced colorectal neoplasia which included malignant and some high risk colorectal lesions that need therapeutic interventions should prevent progression and worse outcomes in the affected cases.

Colonoscopy is one of the cancer screening tests that achieves goals of premalignant and early stage cancer detection and when it is coupled with polypectomy, it can prevent and reduce the incidence of disease, and finally of all results, decrease mortality from colorectal cancer [3–5]. Therefore, colonoscopy is recommended in colorectal cancer screening guideline since 1997 [6]. However, screening colonoscopy is presently not included in the national colorectal cancer screening policy in Thailand nor widely recommended for the Thai population. Most previous studies utilized fecal immunochemical test (FIT) as a first screening tool although the majority of the Thai population do not undergo such testing on a regular basis [7–9]. This study aimed to provide essential information on consideration of implementation of a nation-wide colorectal cancer screening program in Thailand and developing countries. Screening colonoscopy was performed as a first screening tool in all cases while FIT was done as a comparison.

## Methods

### Study participants

After the study protocol had been approved by the Human Research Ethical Committee of Chulabhorn Research Institute, we enrolled participants between 50 and 65 years of age with no past medical history of colorectal cancer or poorly controlled underlying diseases in July 2009. In total, 1,612 applicants expressed their willingness to participate in this project. All participants were educated about colorectal cancer and screening methods that were to be used in this study as well as study objectives, details of each procedure including possible complications, and usage of data from the study. Some patients were excluded based on age, poor medical problem control, or the inability to be followed over a long-term period. Finally, 1,404 participants underwent colonoscopy between July 2009 and June 2010 after they provided informed consent. Their demographic data, medical history, physical examination, and pre-operative management were collected.

### Screening colonoscopy and pathological diagnosis

Bowel preparation before colonoscopy was established by using 90 mL of sodium phosphate after a low fiber diet for 2 days. All participants underwent colonoscopy under intravenous anaesthesia. The colonoscopic findings in each colonic section were recorded. If abnormal mucosal findings or polyps were found, the removed tissue was sent for pathological diagnosis. We categorized abnormal tissue from colonoscopic findings and pathological reports into malignant, high-risk adenoma, low risk adenoma, and non-adenomatous polyps, for which each group underwent different appropriate management according to National Comprehensive Cancer Network (NCCN) guidelines for colorectal cancer screening. High-risk adenoma cases recommended to have repeat colonoscopy earlier than low risk adenoma cases included one of the following colonoscopic and pathological criteria: size ≥1 cm, ≥3 adenomas, or tubulovillous or villous adenoma or high grade dysplasia. All malignant cases were diagnosed by pathological staging using the Seventh edition of the Union for International Cancer Control (UICC) TNM Classification of Malignant Tumours [10]. Patients with colorectal cancer were treated according to the NCCN Guidelines [11]. Participants without malignancy were seen annually in the clinic for general evaluation, and FIT and follow-up colonoscopy was to be performed based on clinical findings and colorectal cancer risks. The second screening colonoscopy was respectively performed at 3, 4, and 5 years follow-up for high risk adenoma cases, low risk adenoma/hyperplastic polyp cases, and normal colonoscopy cases.

### Screening fecal immunochemical test (FIT)

FIT was performed in parallel to screening colonoscopy by fecal occult blood (FOB) one-step test device, a rapid chromatographic immunoassay (Abon Biopharm Hangzhou, China) with relative sensitivity of 93.6 % and relative specificity of 99.1 % [12, 13]. The FOB one-step test can detect fecal blood as low as 50 ng/mL or 6 μg/g feces and is specific for human hemoglobin at a concentration of 1.0 mg/mL. Fecal specimens were self-collected from the first bowel movement in the morning of the appointed date for FIT test which occurred before the start of bowel preparation for screening colonoscopy. The specimens were stored in a clean and dry container and brought in to the hospital by the participants at environmental temperature. The participants were instructed to hand in the samples to the laboratory within 6 h of collection.

### Statistical analysis

Statistical analyses were performed with STATA version 12.1. Data of the participants were reported as means and standard deviation for continuous variables and as proportions and absolute counts for categorical variables. We also estimated FIT sensitivity, specificity and predictive values in detecting colorectal cancer.

## Results

### Demographic characteristics of the study cohort

Of the 1,404 participants, there were 429 males and 975 females, with a mean age of 56.9 ± 4.2 years. Most (81.8 %) of the participants lived in Bangkok Metropolitan and connective territorial provinces during the time of the colonoscopy. Overall, 96.1 % of participants had no lower gastrointestinal symptoms, i.e. bowel habit changes or lower gastrointestinal bleeding or decreased stool calibre or anemia, and the majority (91.7 %) did not have a family history of colorectal cancer, respectively (Table 1). Complete colonoscopy was accomplished in 99.6 % (1,399) of cases. In five incomplete colonoscopy cases, patients were further investigated by CT colonography, all of which produced normal findings.

**Table 1:** Demographic data of the study cohort (*N* = 1,404)

Variable	*N* (%)
Sex	
Male	429 (30.6)
Female	975 (69.4)
Age	56.9 ± 4.2
BMI	
> 25	640 (45.6)
≤ 25	764 (54.4)
Area	
Bangkok and territories	1148 (81.8)
Other	256 (18.2)
Lower gastrointestinal tract symptoms	
Yes	55 (3.9)
No	1349 (96.1)
Family history of colorectal cancer	
Yes	117 (8.3)
No	1287 (91.7)

### Screening colonoscopy findings

**Fig. 1:**
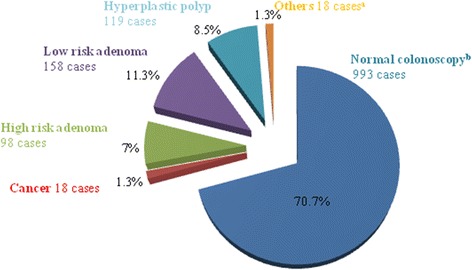
Pathological diagnosis from screening colonoscopy; ^a^including inflammatory polyp, colitis, lipoma and loss specimens; ^b^including diverticulosis without biopsied tissues and hemorrhoids

**Fig. 2:**
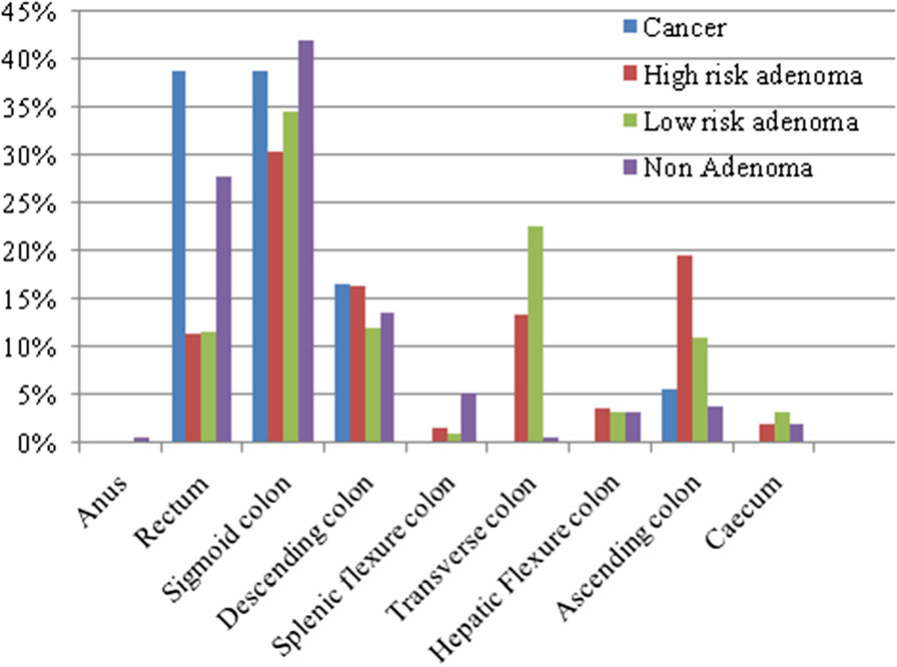
Anatomical site distribution of abnormal colonic tissue from screening colonoscopy

About 30 % (411 cases) of all colonoscopies had abnormal colonoscopic findings. Malignancy was found in 1.3 % (18 cases) of all cases. Of all colonoscopies, 18.2 % (256 cases) had adenomatous polyps, of which 7 % (98 cases) were high risk adenoma. The other 9.8 % (137 cases) had abnormal tissue, of which 8.5 % (119 cases) were hyperplastic polyps (Fig. 1). The pathological diagnosis of malignant tissue in this study showed two cases of carcinoid tumor out of 18 cases. Others were adenocarcinoma with stages varying from stage 0 to stage 4 (Table 2). Nearly 90 % (16 cases) were non-metastatic colorectal cancer; five stage 0 cases, seven stage I cases, and four stage IIA cases. Only two cases from our study had metastasis: one to regional lymph nodes (stage IIIB) and another to other organs (stage IVA). Most abnormalities that required tissue diagnosis were found in the left side of the colon (Fig. 2). The most common cancer site was rectum (7 cases, 38.9 %) and sigmoid colon (7 cases, 38.9 %). Also, the most common site for high risk adenoma and low risk adenoma was sigmoid colon (30 % and 35 %, respectively) and the second most common sites were ascending and transverse colon. Most adenoma had only low grade dysplasia. Only one case of high grade dysplasia was detected in a tubulovillous adenoma case. The gender proportion in 256 cases of adenoma group was 117 males (45.7 %) and 139 females (54.3 %). For colorectal cancer cases, they were 8 males (44.4 %) and 10 females (55.6 %).

**Table 2:** Staging and 5-year survival rates of 18 colorectal cancer cases diagnosed by screening colonoscopy

Variable	*N* (%)
Screening colonoscopy	
Cancer	18 (100)
Stage	
0	5 (27.8)
I	7 (38.9)
IIA	4 (22.2)
IIIB	1 (5.6)
IVA	1 (5.6)
5-year survival rate	15 (83.3)^a^

^a^One case died from unrelated hepatobiliarycancer

Table 3 summarizes histopathology findings of 55 cases with complaining symptoms that may have suggested cancer at the outset of screening and 117 cases with a family history of colorectal cancer. Among 55 cases, 3 developed colorectal cancer (5.5 %) as compared with 15 cases in asymptomatic cases (1.1 %) (*p* = 0.031). Various types of adenomas and polyps were also found in symptomatic cases. If symptomatic cases were excluded, high risk adenoma and colorectal cancer accounted for 8.15 % (110/1,349 cases). Four colorectal cancer cases developed in participants with a family history of colorectal cancer (3.4 %) whereas 14 colorectal cancer cases (1.1 %) were found in a no family history group (*p* = 0.056). Hyperplastic polyps tended to be more common in participants with a family history of colorectal cancer.

**Table 3:** Histological comparison between cases with or without symptoms at screening day 0 visit and between cases with or without family history of colorectal cancer

	Symptoms that may suggest colorectal cancer at the outset			Family history of colorectal cancer		
	Yes 55 cases	No 1,349 cases	*P*-value	Yes 117 cases	No 1,287 cases	*P*-value
Screening colonoscopy						
Colorectal cancer	3 (5.5)	15 (1.1)	0.031^b^	4 (3.4)	14 (1.1)	0.056^b^
Stage						
0	1	4		2	3	
I	0	6		0	6	
IIA	1	4		1	4	
IIIB	0	1		1	0	
IVA	1	0		0	1	
High risk adenoma	3 (5.5)	95 (7.0)		0 (0.0)	98 (7.6)	
Low risk adenoma	10 (18.2)	148 (11.0)		13 (11.1)	145 (11.3)	
Hyperplastic polyp	3 (5.5)	116 (8.6)		14 (12.0)	105 (8.2)	
Other pathological diagnosis^a^	0 (0.0)	18 (1.3)		2 (1.7)	16 (1.2)	
No colorectal tumor	36 (65.5)	957 (70.9)		84 (71.8)	909 (70.6)	

^a^Inflammatory polyp, colitis, and lipoma

^b^Fisher Exact Test

### Sensitivity and specificity of FIT in diagnosis of colorectal cancer

As shown in Table 4, 10/18 colorectal cancer cases had positive FIT whereas 8 colorectal cancer cases were FIT-negative. The sensitivity and specificity of FIT in detection of colorectal cancer was 55.6 % and 96.2 %, respectively, while the positive predictive value (PPV) was 16.4 % and the negative predictive value (NPV) was 99.4 %. Table 5 shows the distribution of FIT-positivity among participants with various histopathology findings. Most of adenoma cases were FIT-negative. FIT-positive non-cancer cases included6 high risk adenoma, 3 low risk adenoma, 8 hyperplastic polyps, and 33 cases with normal colonoscopy results.

**Table 4:** Diagnostic value of FIT in the diagnosis of colorectal cancer

FIT	Colonoscopy	
	Malignancy	No malignancy^a^
Positive	10	51
Negative	8	1,295
Total	18	1,346

^a^FIT not done in 40 cases

Sensitivity (55.6 %), Specificity (96.2 %), PPV (16.4 %), NPV (99.4 %)

**Table 5:** Histopathology characteristics of cases with FIT-positive and FIT-negative results

Histopathology	FIT^a^	
	Positive *N* (%)	Negative *N* (%)
Colorectal cancer	10 (16.4)	8 (0.6)
High risk adenoma	6 (9.8)	87 (6.7)
Low risk adenoma	3 (4.9)	152 (11.7)
Hyperplastic polyp	8 (13.1)	106 (8.1)
Other pathological diagnosis^b^	1 (1.6)	17 (1.3)
No colorectal tumor	33 (54.1)	933 (71.6)

^a^FIT not done in 40 cases

^b^Inflammatory polyp, colitis, lipoma

### Follow-up of study participants and survival of colorectal cancer cases

In the follow-up period after colonoscopic screening, there were three additional new cases of colorectal cancer. Two cases of intramucosal carcinoma of sigmoid colon and another case with stage 1 anal canal adenocarcinoma were found in the second year from the group previously identified as high risk adenoma, low risk adenoma and hyperplastic polyp, respectively. The mortality due to colorectal cancer cases in this study was 3/18 cases: 1 case with stage IV disease died in the first year, 1 case with stage IIA in the fourth year, and another case in stage I died from unrelated primary hepatobiliary cancer. The overall survival of colorectal cancer cases at 5 year was 83.3 % (88.9 % if excluded 1 death from unrelated cancer).

## Discussion

The discovery rate of high risk adenoma and invasive colorectal cancer in this study (8.15 %) was nearly twice that of advanced colorectal neoplasia in the asymptomatic population (4.5 %) as reported from multiple studies in Asia but comparable to that of the symptomatic population (7.8 %) [14]. These patients should receive therapeutic intervention before progression to more advanced disease. When we excluded the amount criteria from the high risk adenoma group, the prevalence of advanced colorectal neoplasia in our study (3 %) and also adenocarcinoma (1.3 %) were still comparable to the country with a high incidence of colorectal cancer [14, 15]. Prior hospital-based reports and retrospective studies from Thailand also showedcancer detection rate by colonoscopy of 0.6–7.1 % in different studied population [7–9, 16, 17]. These previous studies utilized FIT as a first screening tool and were unlike our study whereby colonoscopy was performed as a first screening tool.

The abnormal colonic tissue (29.3 %) findings from our screening colonoscopy study are in the range of polyp detection rates (25–37 %) reported in other studies from within and outside Thailand [16–20]. Both adenomatous polyp and hyperplastic polyposis can accumulate worse genetic changes and become malignant [21, 22]. Therefore, colorectal cancer screening would benefit this population. Although most abnormalities that required tissue diagnosis were found in the left side of the colon, complete colonoscopy should be done to include low and high risk adenomas in the second most common locations, the transverse and ascending colon.

There are some key differences in the selection of subjects for our study compared with others: our study included screening criteria for an age group at high risk of colorectal cancer [23], while others included data from a symptom-related group. Moreover, this result might not represent the incidence of colorectal cancer from all regions in Thailand because most (81.8 %) of the participants were from the Bangkok metropolitan region and territories. If this finding represented the real incidence of colorectal cancer in our population, or perhaps even in urban and suburban areas, launching a national colorectal cancer screening policy should be considered for specific groups. Other factors that might affect a high yield of colorectal cancer findings include public information that may stimulate awareness and access to reluctant cancer patients.

Problems related to colonoscopy screening include its invasiveness, complications, high financial cost, and need of specialized endoscopists; these reasons were reported as causes of low colorectal screening adherence in well developed countries [24, 25]. Therefore, other more practical screening methods were utilized instead of colonoscopy. The sensitivity of FIT for cancer detection in our study (55 %) was comparable to Guaiac fecal occult blood test (37–79 %) but somewhat lower than results of FIT in other studies (79 %) [26, 27]. In some resource-limited countries, fecal-based screening may play a major role in decreasing colorectal cancer mortality with high subject acceptance rates [28, 29]. However, in our study, the sensitivity of FIT of 55.6 % was deemed inadequate to detect colorectal cancer cases and 8/18 cancer cases had negative FITs.

There are no recommendations for any other surveillance procedure after screening colonoscopy, but endoscopic management after colonoscopic and pathologic findings have been suggested [30–34]. However, in this study, cases of colorectal cancer were found before the recommended period of surveillance colonoscopy, which were similar to the findings of other studies [5, 24, 35–39]. Moreover, some cases also suffered from unrelated types of cancer. Thus, some individual risk criteria such as smoking, a family history of biliary tract cancer which are common in Thailand should be concerned for improvement of screening benefits in specific groups of patients [40, 41]. Quality indices [42, 43], technology of colonoscopy [44] and other predictors [45–48] should be included for detection of these interval cancers. Particularly, with the aging population in Thailand, proper screening and surveillance programsneed to be further evaluated [3].

The mortality caused by colorectal cancer in our study after monitoring for 5 years was only two cases from colorectal cancer. Another case died from unrelated hepatobiliary cancer. So the 5-yearsurvival rate after screening colonoscopy and standard treatment of detected colorectal cancer cases in this study, even including five cases (27.8 %) with stage IIA and IIIB, was excellent at 88.9 %. This survival rate was comparable to survival of stage I colorectal cancer (87–92 %) after standard staging and treatment [49].

## Conclusions

In our study population aged 50–65 years old, 30 % of colonoscopically screened cases had abnormal colonic lesions that needed further pathological diagnosis. The colorectal cancer detection rate was 1.3 % and the overall prevalence of advanced colorectal neoplasia was 3 % by screening colonoscopy. Tissue abnormalities were found more in the distal part than the proximal part of the large intestine. The overall survival of colorectal cancer patients diagnosed by colonoscopic screening was 83 %. FIT was inadequate to detect advanced colorectal neoplasia and missed almost half of the colorectal cases. We suggest that screening colonoscopy should be implemented as part of the National Colorectal Cancer Screening Program in Thailand.

## Abbreviations

BMI: Body mass index
CT colonography: Comperized tomographic colonography
FIT: Fecal immunochemical tests
FOB: Fecal occult blood
NCCN: National Comprehensive Cancer Network
NPV: Negative predictive value
PPV: Positive predictive value
UICC: The Union for International Cancer Control
